# Effects of stellate ganglion block on postoperative trigeminal neuropathy after dental surgery: a propensity score matching analysis

**DOI:** 10.1038/s41598-020-70533-w

**Published:** 2020-08-10

**Authors:** Teppei Sago, Osamu Takahashi, Mika Ogawa, Kazune Kawabata, Ibuki Matsukawa, Shunji Shiiba

**Affiliations:** 1 Division of Dental Anesthesiology, Department of Science of Physical Functions, Kyushu Dental University, 2-6-1, Manazuru, Kokura-kita, Kitakyushu, Fukuoka 803-8580 Japan; 2 Division of Oral and Maxillofacial Surgery, Department of Science of Physical Functions, Kyushu Dental University, Kitakyushu, Japan; 3 Section of Anesthesiology, Department of Diagnostics and General Care, Fukuoka Dental College, Fukuoka, Japan

**Keywords:** Outcomes research, Dentistry

## Abstract

This study aimed to evaluate the effects of stellate ganglion block (SGB) on postoperative trigeminal neuropathy (TNP) after dental surgery. This was a retrospective study based on the medical records of all patients with postoperative TNP at Kyushu Dental University Hospital from 2014 to 2019. Patients were divided into the SGB group (received SGB) and non-SGB group (did not receive SGB). We evaluated the severity of TNP at 3 months after surgery and the incidence rate of abnormal sensations. Abnormal sensations were counted using patients’ reports of uncomfortable symptoms during the treatment, including dysaesthesia, allodynia, and hyperalgesia. A propensity score (PS) matching analysis was performed to evaluate these data. After PS matching, amongst others, the force equivalent values of the Semmes–Weinstein test at 3-months post-treatment were significantly lower in the SGB group than in the non-SGB group (2.00 ± 0.44 vs 2.30 ± 0.48; p < 0.05). In addition, after PS matching, the incidence rate of abnormal sensations during the treatment was significantly lower in the SGB group than in the non-SGB group (10 cases [4.7%] vs 22 cases [10.3%]; p < 0.05). Collectively, the findings support that SGB may improve the recovery from postoperative TNP and reduce the incidence rate of abnormal sensations after dental surgery.

## Introduction

Postoperative trigeminal neuropathy (TNP) is a common complication after dental surgery^1–4^. Although neuropathic pain (NPP) after dental surgery is uncommon, the risk of severe nerve paralysis or NPP does exist and can decrease patient satisfaction^5–8^. According to previous studies, especially those that assessed recovery of the inferior alveolar nerve after sagittal split ramus osteotomy (SSRO), nerve damage was the most striking during the first 3 months after surgery^9,10^. Another study reported that patients with a persistent trigeminal nerve deficit have poorer health-related and oral health-related quality of life than do those without such a deficit^11^.

Treatments for postoperative TNP have been suggested, including oral vitamin B12 (VB12) and adenosine triphosphate, xenon light irradiation, and stellate ganglion block (SGB)^12^, none of which have been proven effective. Although SGB is a commonly used technique for the treatment of various diseases and conditions^13^, including peripheral neurosensory disturbances^14^, the efficacy of SGB for TNP is poorly documented. It would be unethical to conduct a randomised controlled trial (RCT) for postoperative TNP because postoperative TNP is an undesirable outcome for patients. Furthermore, it is difficult to compare the treatment efficacy of SGB with that of other treatments in an observational study because the treatment of nerve paralysis is determined by various factors, including the severity of paralysis and patient characteristics.

Propensity score (PS) analysis is a statistical method to adjust for confounding by indication (CI), which is a major problem in the comparative analysis of treatment effects using observational data^15,16^. CI represents the bias through which the patients’ characteristics and factors related to the medical facility affect not only the treatment outcomes but also the treatment allocation. Although an RCT is the best method for eliminating CI, the effects of CI can be overcome, to some extent, by performing a PS matching analysis using observational data.

In this study, we aimed to investigate the efficacy of SGB for postoperative TNP by performing a PS matching analysis using observational data.

## Methods

This study was approved by the Institutional Review Board of Kyushu Dental University (approval number: 19-84), and all experiments were performed in accordance with relevant guidelines and regulations. Informed consent for a retrospective review was obtained from the patients before treatment.

### Study population

From January 2014 to December 2019, 751 patients were diagnosed with postoperative TNP at Kyushu Dental University Hospital. Demographic and clinical data of the enrolled patients were retrospectively collected from their medical records. Amongst the 751 patients who underwent postoperative TNP, 77 were excluded from this study for the following reasons: steroid users (n = 8), suspected neurotmesis (n = 2), patients in whom the initial assessment were conducted more than 1 month after surgery (n = 15), and incomplete medical records (n = 52). A total of 674 patients were included in this study. Patients who received SGB were defined as the SGB group, and the remaining patients were defined as the non-SGB group.

### Covariates

Covariates included age, sex, type of surgery (tooth extraction, Le Fort I osteotomy + SSRO, SSRO, dental implant placement), type of nerve (maxillary nerve, mandibular nerve), type of treatment (SGB, non-SGB), time from surgery to treatment initiation, severity of nerve paralysis before treatment and after 3 months of treatment, and incidence of abnormal sensations. Treatments for the non-SGB group included oral adenosine triphosphate and xenon light irradiation.

The severity of nerve paralysis was assessed by the electric detective threshold (EDT), current perception threshold (CPT), and Semmes–Weinstein (SW) tests. The EDT was measured using an electrodiagnostic device called a two-channel universal stimulator NS-101 (Unique Medical, Tokyo, Japan). EDT measurements were performed as follows: under a rectangular wave current of 100 ms, 3 Hz was applied to the affected region, increasing the current strength by 0.1 mA per second, with the strength recognised by the patient defined as the EDT^17^. The CPT was measured using an electrodiagnostic device called a Neurometer (Neurotron, Baltimore, MD, USA), which has been applied successfully for the objective quantification of peripheral nerve function in previous studies^18,19^. The affected site received three stimulus frequencies that are known to stimulate the Aβ (2,000 Hz), Aδ (250 Hz), and C-fibres (5 Hz). The SW test was performed using a SW monofilament set (Sakai Medical, Tokyo, Japan). The SW monofilament set has a filament length of 38 mm and 20 different diameters. As the filament diameter increases, the delivery force gradually increases. A three-digit number that reflected the common log of the force measured in tenths of a milligram was used to represent each diameter. Measurements were performed according to the methods described in previous studies^20,21^. Abnormal sensations were counted using patients’ reports of the uncomfortable symptoms they experienced during the treatment, including dysaesthesia, allodynia, and hyperalgesia.

### Outcomes

The primary outcomes were the EDT and CPT values, as well as the force equivalent values of the SW test, after 3 months of continuous treatment. The secondary outcome was the incidence rate of abnormal sensations.

### Statistical analysis

PS matching was used to compare the outcomes between the SGB and non-SGB groups^20^. A multivariable logistic regression model was used to predict the patients’ PS for SGB. Possible confounders were selected for their potential association with the outcomes of interest based on clinical knowledge. Predictor variables included age, sex, type of surgery, type of nerve, time from surgery to treatment initiation, and severity of nerve paralysis before treatment. One-to-one nearest neighbour matching without replacement was conducted for the estimated PS of the patients and the calliper width was set to 20% of the standard deviation for the PS^22,23^. To evaluate the matching performance, absolute standardised differences were used to compare the baseline characteristics before and after PS matching. If the absolute standardised difference was 10% or less, imbalances between the two groups were considered negligible^24^. In addition, the Mann–Whitney *U* test and Fisher’s exact test were used to compare the EDT values, CPT values, and force equivalent values of the SW test at 3 months after treatment, as well as the presence of abnormal sensations, respectively, between the SGB and non-SGB groups after PS matching.

Continuous variables are presented as means ± standard deviations, and categorical variables are described as numbers (%). A two-sided p value of < 0.05 was considered to indicate statistical significance. PS matching and all statistical analyses were performed using SPSS (version 25.0; IBM, Chicago, IL, USA).

## Results

**Figure 1:**
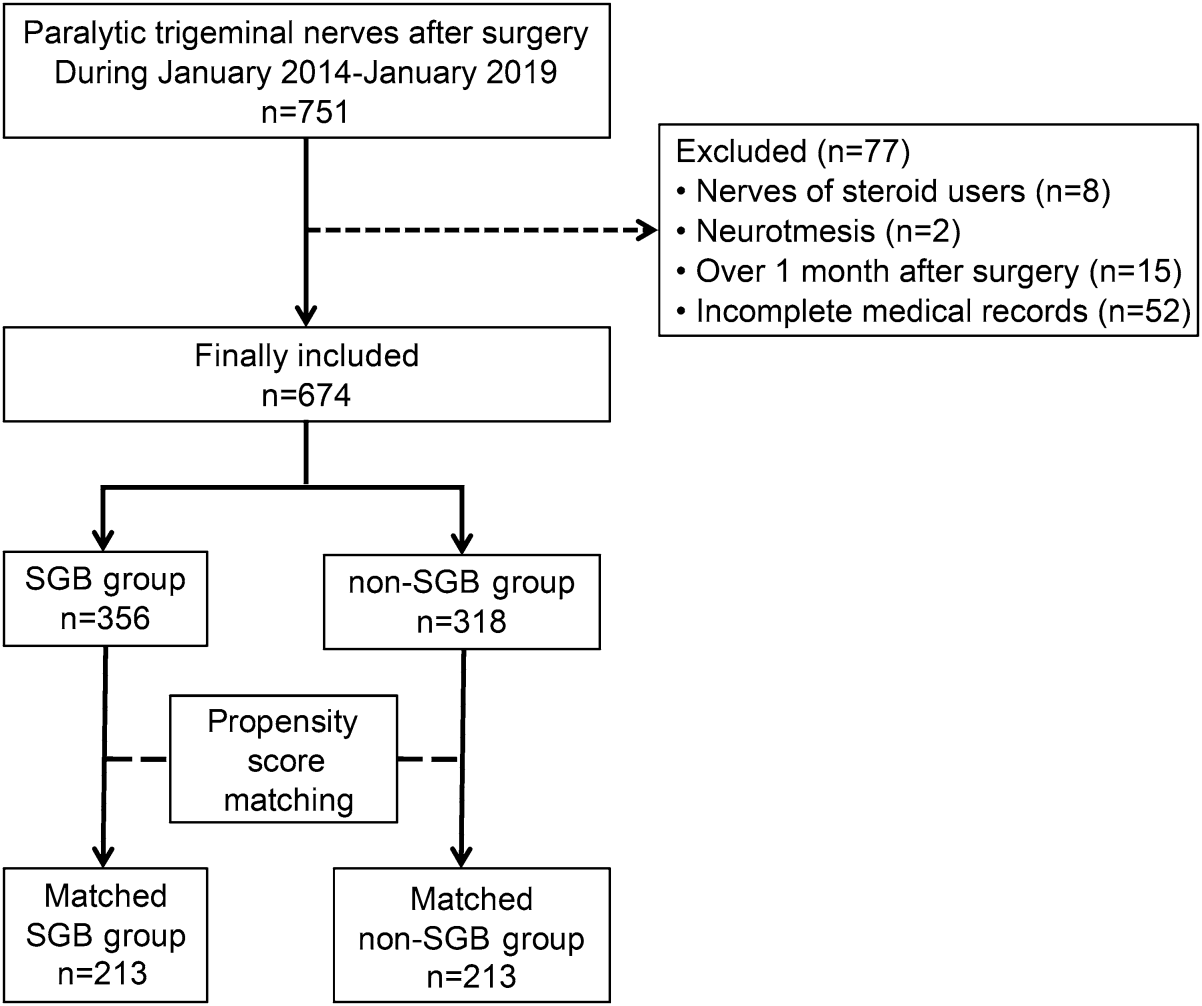
Flowchart representing the patient selection process for the study. The study focused on 751 patients who were diagnosed with nerve paralysis. The patients were divided into two groups for analysis: 356 patients in the SGB group and 318 patients in the non-SGB group. To control for selection bias, a comparative study was performed in 426 patients using one-to-one propensity score matching (213 patients in the SGB group, 213 patients in the non-SGB group). *SGB* stellate ganglion block.

We identified 751 patients who were diagnosed with postoperative TNP from January 2014 to January 2019 (Fig. 1). A total of 674 patients met the inclusion criteria, including 356 who received SGB and 318 who did not. All patients with neurosensory deficits received VB12 before SGB or other treatments, and the VB12 was continued for at least 3 months.

Before PS matching, there were significant differences in age, sex, type of surgery, type of nerve, time from surgery to treatment initiation, and severity of nerve paralysis before treatment between the SGB and non-SGB groups (Table 1). All variables were well balanced after PS matching (Table 1).

**Table 1:** Patient characteristics before treatment.

	Before PS matching			After PS matching		
	SGB (n = 356)	Non-SGB (n = 318)	Absolute standardised difference (%)	SGB (n = 213)	Non-SGB (n = 213)	Absolute standardised difference (%)
Age (years)	33.39 ± 13.08	34.99 ± 16.26	11	33.51 ± 12.53	34.92 ± 16.42	9
**Sex**						
Male	105 (29%)	91 (28%)	2	60 (28%)	69 (32%)	9
Female	251 (71%)	227 (72%)	2	153 (72%)	144 (68%)	9
**Time to treatment (days)**	6.73 ± 2.12	7.19 ± 1.81	23	6.92 ± 2.35	7.07 ± 1.44	8
**Type of nerve**						
Maxillary	71 (20%)	39 (12%)	22	28 (13%)	30 (14%)	3
Mandibular	284 (80%)	280 (88%)	22	185 (87%)	183 (86%)	3
**Type of surgery**						
SSRO	127 (36%)	131 (41%)	10	88 (41%)	92 (43%)	4
SSRO + Le Fort I	132 (37%)	54 (17%)	47	57 (27%)	51 (24%)	7
Extraction	94 (26%)	130 (41%)	32	67 (31%)	68 (32%)	3
Implant	3 (1%)	3 (1%)	0	1 (1%)	2 (1%)	0
**EDT value before treatment (mA)**	1.74 ± 0.62	1.42 ± 0.45	59	1.59 ± 0.61	1.57 ± 0.45	4
**CPT value before treatment (μA)**						
2000 Hz	83.52 ± 44.20	66.45 ± 29.00	45	75.70 ± 43.11	74.79 ± 29.30	3
250 Hz	52.12 ± 22.96	42.92 ± 21.23	42	48.13 ± 22.38	48.16 ± 21.99	0
5 Hz	64.72 ± 32.80	51.37 ± 23.25	47	59.19 ± 33.95	57.80 ± 23.49	5
**SW test before treatment**	3.65 ± 0.76	3.11 ± 0.79	70	3.40 ± 0.68	3.41 ± 0.78	1

Data are presented as number of patients (percentage) or mean ± standard deviation.

*PS* propensity score, *SGB* stellate ganglion block, *SSRO* sagittal split ramus osteotomy, *Le Fort I* Le Fort type I osteotomy, *EDT* electric detective threshold, *CPT* current perception threshold, *SW* Semmes–Weinstein.

After PS matching, the EDT value, the stimulus frequencies of the CPT, and the force equivalent values of the SW test at 3 months after treatment were significantly lower in the SGB group than in the non-SGB group (Table 2).

**Table 2:** Comparison of outcomes between the two groups at 3 months after treatment.

	Before PS matching			After PS matching		
	SGB (n = 356)	Non-SGB (n = 318)	p value	SGB (n = 213)	Non-SGB (n = 213)	p value
**EDT value after treatment (mA)**	0.95 ± 0.41	0.89 ± 0.28	0.49	0.76 ± 0.21	0.97 ± 0.25	< 0.001
**CPT value after treatment (μA)**						
2000 Hz	41.41 ± 18.12	38.67 ± 13.70	0.66	33.71 ± 10.43	40.62 ± 12.05	< 0.001
250 Hz	27.87 ± 14.99	25.17 ± 10.67	0.51	21.33 ± 7.86	26.59 ± 9.06	< 0.001
5 Hz	32.99 ± 16.03	29.78 ± 11.50	0.32	26.17 ± 7.83	32.35 ± 9.73	< 0.001
**SW test after treatment**	2.34 ± 0.80	2.16 ± 0.51	0.28	2.00 ± 0.44	2.30 ± 0.48	< 0.001

Data are presented as mean ± standard deviation.

*PS* propensity score, *SGB* stellate ganglion block, *EDT* electric detective threshold, *CPT* current perception threshold, *SW* Semmes–Weinstein.

After PS matching, the incidence rate of abnormal sensations experienced by patients during the treatment was significantly lower in the SGB group than in the non-SGB group (Table 3).

**Table 3:** Comparison of the incidence rate of abnormal sensations between the two groups.

	Before PS matching			After PS matching		
	SGB (n = 356)	Non-SGB (n = 318)	p value	SGB (n = 213)	Non-SGB (n = 213)	p value
Abnormal sensation (+)	34 (9.6%)	31 (9.7%)	n.s	10 (4.7%)	22 (10.3%)	0.042
Abnormal sensation (−)	322 (90.4%)	287 (90.3%)		203 (95.3%)	191 (89.7%)	

Data are presented as number of patients (percentage).

*PS* propensity score, *SGB* stellate ganglion block, *n.s*. not significant.

## Discussion

This study investigated the effects of SGB on postoperative TNP after dental surgery while adjusting for patient characteristics, type of surgery, type of nerve, time from surgery to treatment initiation, and the severity of nerve paralysis before treatment. The results showed that SGB was associated with better recovery and a slight reduction in the incidence of abnormal sensations in patients with postoperative TNP.

Previous studies investigating the effects of SGB on TNP had small sample sizes, short study periods, and included only a few surgery types^12,14^. Relative to such prior studies, the strengths of the current study are its larger sample size, longer study period, and use of additional surgery types. Furthermore, the number of propensity-matched patients in this study (n = 426) was large enough to demonstrate the significant effects of SGB for TNP.

In this study, the severity of nerve paralysis after 3 months was a better predictor of recovery in the SGB group than in the non-SGB group. Although the association between SGB and recovery of TNP has not been well investigated, previous animal studies have demonstrated various effects of SGB. In a rabbit model, SGB increased the blood flow in the craniocervical region^25^ and changed the in-tissue oxygen tension^26^. In a rat model, SGB changed the blood flow at the mandibular angle^27^, and a sympathetic nerve block at an early stage promoted neurophysiological recovery and regeneration of damaged infraorbital nerves^28^. In humans, SGB increased the blood supply to the head, neck, and upper limbs as a result of vasodilatation^29^. Based on these mechanisms, it is likely that SGB promotes nerve regeneration and neurological recovery, thereby improving the clinical outcomes of postoperative TNP.

Our findings indicate that SGB reduced the incidence of abnormal sensations, including dysaesthesia, allodynia, and hyperalgesia, during the 3 months following surgery. Patients with NPP often complain of sensory loss and experience so-called positive phenomena, including spontaneous pain, dysaesthesia, allodynia, hyperalgesia, and hyperpathia^30^. Our results suggest that SGB can reduce the incidence of NPP after dental surgery. This may be biologically plausible because of an association between the sympathetic nervous system and the development of NPP. Previous studies have suggested that the therapeutic mechanism of SGB is to block the sympathetic nerves of the head, neck, and upper limbs^29,31^, whilst the analgesic mechanism of SGB could be to block the neuronal connections of the sympathetic nerves^32^. Moreover, some studies have shown that SGB is effective for reducing sympathetically mediated pain in the orofacial region^33–35^. The present results demonstrating a lower incidence of abnormal sensations in the SGB group than in the non-SGB group are consistent with these proposed mechanisms and therapeutic effects of SGB.

Several limitations of this study should be noted. First, due to the retrospective and observational nature of the study, potential bias and confounding were not completely eliminated. Although all measured variables were well balanced, we could not adjust for unmeasured potential confounders such as vital signs, operative time, and haemorrhage volume in surgery because these variables were not always included or fully described in the patients’ medical records. Second, it is likely that a period of 3 months was too short to assess the incidence of NPP because the onset of NPP after the initial injury may be delayed by any amount of time, from days to several months^5^. Finally, some of the abnormal sensations abstracted from medical records in this study were not completely suited to the symptoms of NPP, including spontaneous pain, dysaesthesia, allodynia, hyperalgesia, and hyperpathia.

## Conclusion

In this study, SGB was associated with better recovery and a reduced incidence of NPP amongst patients with postoperative TNP after dental surgery. The results of this study suggest that SGB is an effective therapy for TNP after dental surgery. Despite being an observational study, our study provides a treatment option for TNP after dental surgery and underscores the need for further investigations and future multi-centre studies on this topic.

## Data Availability

The dataset used in this study is available from the corresponding author on reasonable request.
